# Awareness and Early Diagnosis of Popliteal Artery Entrapment Syndrome Is Needed∗

**DOI:** 10.1016/j.jaccas.2022.02.002

**Published:** 2022-04-06

**Authors:** Jihad A. Mustapha, Rajabrata Sarkar, Ujjwal Rastogi

**Affiliations:** a Advanced Cardiac and Vascular Centers for Amputation Prevention, Grand Rapids, Michigan, USA; b Department of Medicine, Michigan State University College of Human Medicine, East Lansing, Michigan, USA; c Division of Vascular Surgery, Department of Surgery, University of Maryland School of Medicine, Baltimore, Maryland, USA; d Department of Medicine, Louisiana State University, Cardiovascular Institute of South, Lafayette, Louisiana, USA

**Keywords:** peripheral vascular disease, stents, treatment, ultrasound

**Figure:**
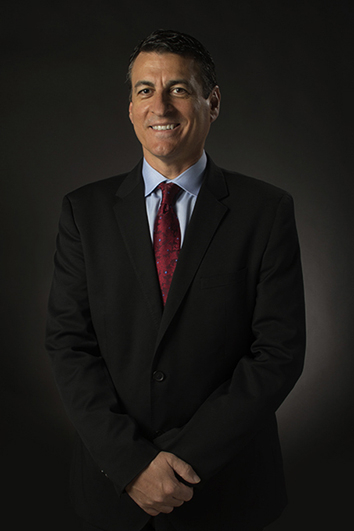

The anatomic basis for popliteal artery entrapment syndrome (PAES) was first described by Stuart in 1879.^1^ However, the clinical condition associated with such anatomic abnormalities was not described until 1958.^2^

PAES describes a group of conditions in which compression of the popliteal artery (POP), popliteal vein, and tibial nerve (singly or in combination) in the popliteal fossa by surrounding musculoskeletal structures occurs to a degree sufficient to cause vascular and neurogenic symptoms.^3^ PAES can be due to a congenital abnormality in the arrangement of the posterior calf musculature and the popliteal vessels (Figure 1)^4^ or, more commonly, can be functional popliteal entrapment from hypertrophy of muscles to occlude a POP that has a normal course. It is important to distinguish anatomic from functional popliteal entrapment because the natural history and clinical consequences of these disorders are different. Many patients with anatomic entrapment will experience progression to occlusion of the POP from sustained distortion, with subsequent severe ischemia. By contrast, patients with functional entrapment have stable claudication with vigorous exercise but have a normal anatomic course of the artery and do not experience arterial damage and occlusion over time.

**Figure 1:**
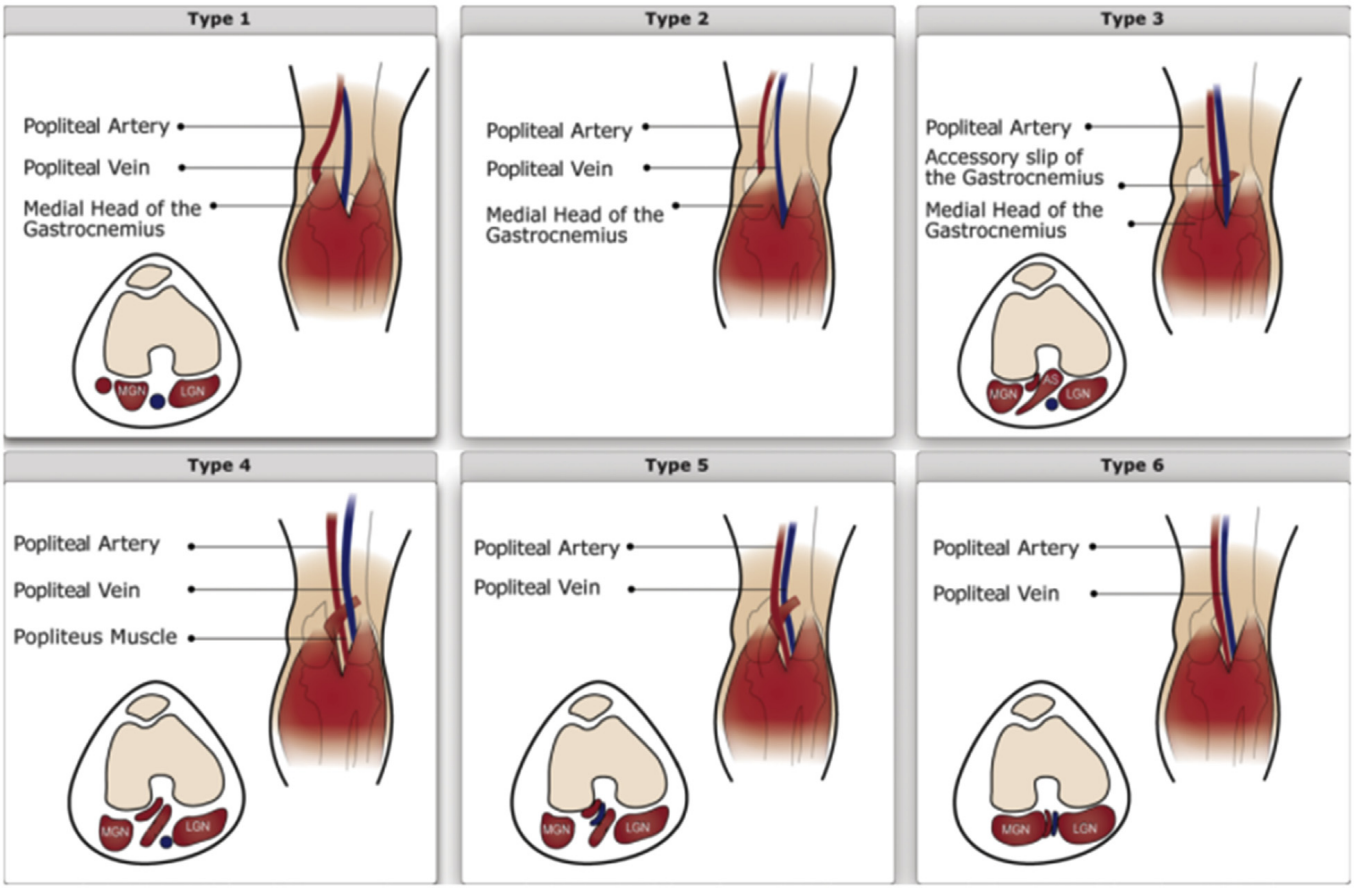
The 6 Types of Popliteal Artery Entrapment Syndrome Schematic drawings showing the 6 types of popliteal artery entrapment syndrome. In type 1, the artery is displaced medially by an abnormal head of the gastrocnemius muscle, which inserts laterally on the distal femur. In type 2, the artery is displaced medially by an abnormal head of the gastrocnemius muscle, which inserts laterally in the distal femur. In type 3, the normally positioned artery is enveloped and entrapped by an aberrant accessory slip from the medial head of the gastrocnemius muscle. In type 4, the artery is entrapped by its location deep in the popliteus muscle or beneath fibrous bands in the popliteal fossa. In type 5, the artery and vein are both entrapped. In type 6, the functional, normally positioned artery is entrapped by a normally positioned but hypertrophic muscle.

The main symptoms of entrapment are pain and cramping in the lower calf during exercise, resolving with rest. Other signs and symptoms are a cold foot after exercise, paresthesia, or numbness in the calf. These are unusual findings in the claudication patient owing to the typical arterial ischemic disease alone. The nerve next to the POP and vein is also compressed, causing these unusual findings. Finally, the vein itself can also get compressed, becoming engorged and leading to a feeling of heaviness in the calf and cramping at night with swelling of the calf and ankle. If this continues for a long time, skin changes may develop. Rarely, deep vein thrombosis occurs.

Functional popliteal entrapment is commonly seen in teenaged and older competitive athletes. Often the patient describes vigorous weight training before the development of symptoms. Most patients have undergone extensive orthopedic evaluation, including compartment pressure testing, the result of which is often positive with functional entrapment and leads to fasciotomy without relief of symptoms.

Anatomic entrapment is seen in patients younger than age 40 who are otherwise healthy. The vascular specialist needs to consider nonatherosclerotic causes of arterial ischemia in this population, including arterioembolism, cystic adventitial disease, and popliteal entrapment.

When patients present with unusual, unilateral, severe claudication and/or occluded superficial femoral artery/POP with or without some tibial occlusions secondary to thromboembolization and otherwise no additional indication of atherosclerosis, one should be suspicious of PAES.

In the paper by Wittig et al^5^ in this issue of *JACC: Case Reports*, the authors describe 2 cases of interwoven nitinol femoropopliteal stent fractures due to PAES. I commend the authors for drawing attention to 2 patients with long-term complications of initially misdiagnosed PAES. Although these cases are rare in the literature, they are not so rare in the real world.

Patients with anatomic popliteal entrapment who experience more severe PAES symptoms later in life often have had milder symptoms ongoing for many years that have either been ignored by the patient or misdiagnosed by health care providers. The symptoms generally progress over time until the patient presents in an urgent fashion, with ischemia at rest from thrombosis or embolization.

Additionally, older adults with no previous symptoms can experience functional PAES when they begin exercising later in life, particularly with activities such as running, biking, and weight training that build muscle mass in the lower extremity, leading to PAES. This is important history to note in the examination of the adult patient who presents with new onset of claudication.

The article very nicely shows, in case 1, an angiogram in a patient with severe claudication without angiographic evidence of advanced atherosclerosis. Fortunately, further functional angiography with the addition of maximal knee extension and dorsal extension of the foot allowed the operators to accurately diagnose PAES in this patient. Physicians should be familiar with the proper maneuvers to confirm absence or presence of PAES.

The physical examination in patients who present with symptoms tends to reveal the underlying cause if the following examination techniques are used. As a patient lies flat on the back, the common femoral artery, POP, dorsalis pedis artery, and posterior tibial artery (PT) should all be examined for the presence of a palpable pulse. Most of the time the common femoral artery and POP pulses will be palpable. The dorsalis pedis artery and PT could be palpable or absent. Either way, one who is looking to rule out PAES will proceed with performing functional palpable examination of the DP and PT. This is performed by placing the index and third fingers at the dorsum of the foot where the DP pulse is expected to be. Regardless of what the examiner finds regarding a palpable DP pulse, the next maneuver in the functional examination is to keep the fingers in the same location and ask the patient to perform both plantar and dorsal flexion of the foot. The DP pulse may disappear or become weak in either position, but rarely in both positions. The same technique is used to examine the PT artery by placing the fingers below the medial malleolus and then asking the patient to perform plantar and dorsal flexions. When the patient performs the plantar and dorsal flexions, the calf muscle, in particular the gastrocnemius muscle, is triggered, which happens to have its heads inserted at the level of the popliteal fossa behind the knee. The isometric contractions cause the muscles to stiffen and bulk up, leading to compression of the POP, which in turns leads to functional dynamic change in the sudden absence of the DP and PT pulses and return of the pulses during the relaxation time of the calf muscles.

Patients with suspected PAES should undergo noninvasive arterial studies, including ankle-brachial index and arterial duplex scanning. Patients with functional PAES will consistently have normal study results at rest. In suspected functional PAES (i.e., young athletic patient with normal noninvasive study results), we recommend: 1) axial imaging with either CT angiogram or MRA, preferably with imaging at rest and with active plantar flexion; 2) ankle-brachial index study with active plantar flexion or post-exercise; and 3) sports medicine consultation to exclude other musculoskeletal diagnoses such as tibial stress fracture, chronic exertional compartment syndrome, or ligamentous pathology of the knee. Axial imaging also allows evaluation of the course of the POP and vein, and determination of any abnormal musculature (e.g., anatomic PAES). Ultimately, we often recommend angiography with intravascular ultrasound done at rest and with active plantar flexion to confirm the diagnosis (Figures 2 and 3)

**Figure 2:**
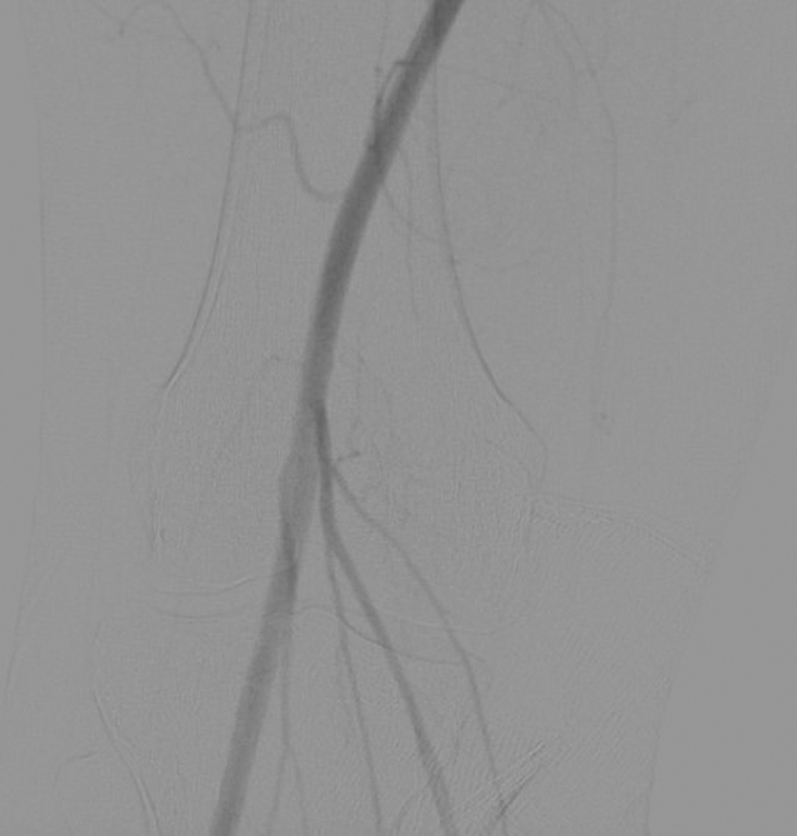
Angiogram of Right Popliteal Artery at Rest and With Active Plantar Flexion

**Figure 3:**
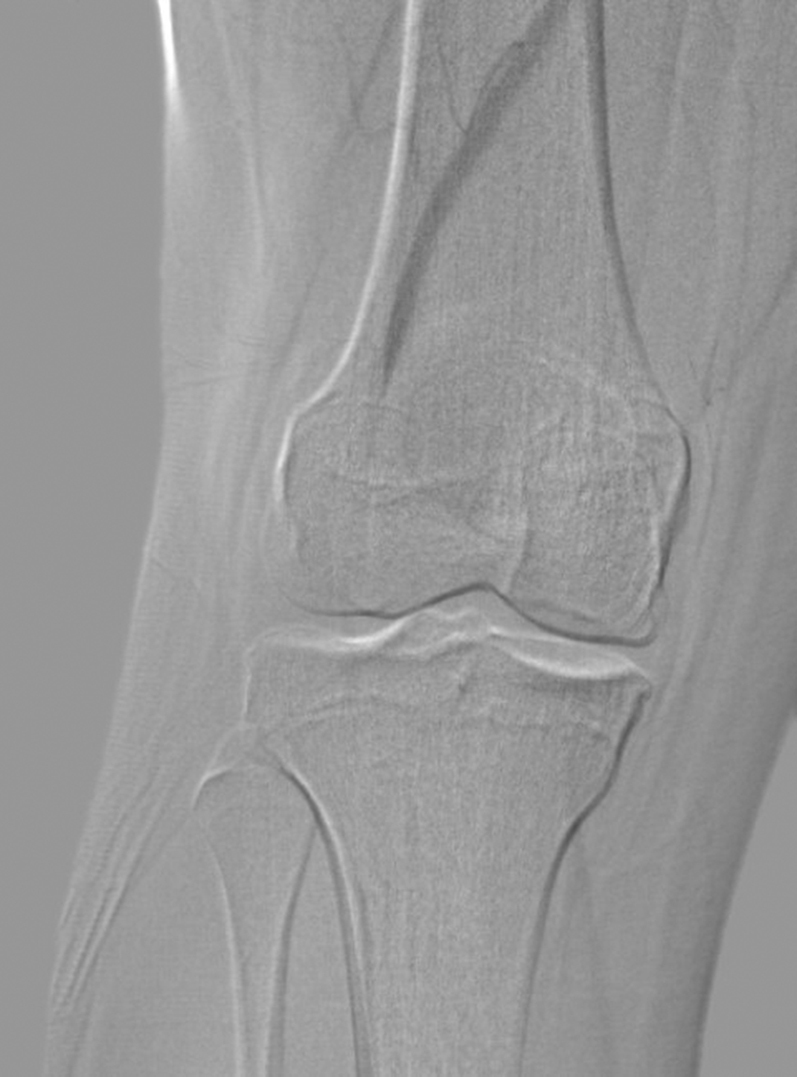
Angiogram of Right Popliteal Artery at Rest in a College Athlete With Functional Popliteal Artery Entrapment Syndrome

In older patients (i.e., >25 years of age), axial imaging is often followed by angiography to diagnose other unusual conditions (e.g., adventitial cystic disease of the POP). Patients with anatomic PAES may present later in life with either arterial occlusion, and this is commonly mistaken for atherosclerotic peripheral artery disease. The inclusion of intravascular ultrasound and active flexion maneuvers during angiography is helpful in establishing a component of PAES in patients with established ischemia.

Treatments of anatomic and functional PAES are different and also depend on the degree of ischemia present. Functional PAES in athletes can be treated with injection of botulinum toxin (Botox) into the gastrocnemius and plantaris muscles, but long-term relief of symptoms is uncommon.^6^ Calibrated surgical resection of the compressing muscles provides durable relief; however, vigorous weight training can cause the compression and symptoms to recur. Patients with anatomic PAES should undergo surgery to correct the course of the POP and resect abnormal muscular structures. Patients with PAES who present with acute arterial ischemia ideally should undergo endovascular removal of the thrombus followed by surgical correction of the PAES. In patients with chronic popliteal occlusion due to PAES (usually anatomic), arterial bypass is often required.

A study performed by Lejay et al^7^ in 2016 showed that surgery performed to treat PAES in 18 patients (25 limbs) with a median age of 35 years had good 5-year outcomes.^7^ This study supports early diagnosis and treatment of this syndrome to prevent long-term complications from PAES. Therefore, awareness must be raised to result in earlier diagnosis and treatment to prevent the long-term complications of nerve, muscle, arterial, and venous damage that are avoidable if PAES is treated early.

## Funding Support and Author Disclosures

The authors have reported that they have no relationships relevant to the contents of this paper to disclose.
